# The Molecular Simulation Study of nNOS Activation Induced by the Interaction Between Its Calmodulin-Binding Domain and SUMO1

**DOI:** 10.3389/fnmol.2020.535494

**Published:** 2020-10-29

**Authors:** Nan Wang, Xiao-Yu Hou

**Affiliations:** ^1^Research Center for Biochemistry and Molecular Biology, Jiangsu Key Laboratory of Brain Disease Bioinformation, Xuzhou Medical University, Xuzhou, China; ^2^State Key Laboratory of Natural Medicines, School of Life Sciences and Technology, China Pharmaceutical University, Nanjing, China

**Keywords:** nNOS/NOS1, SUMO-ylation, molecular dynamic, SUMO1 modification, calmodulin binding domain

## Abstract

Neuronal nitric oxide synthase (nNOS), an enzyme required for learning and memory, catalyzes L-arginine decomposition during nitric oxide production in mammalian neurons. Over-activation of nNOS leads to oxidative/nitrosative stress, which is part of the pathophysiological process of various neuropsychiatric disorders. Previous experimental studies suggest that nNOS is a target for small ubiquitin-like modifier 1 (SUMO1), and that SUMO1-ylation upregulates nNOS catalytic activity in hippocampal neurons. To date, a comprehensive structural model has not been proposed for nNOS SUMO1-ylation. In this study, our aim was to build *in silico* models to identify the non-bonded interactions between SUMO1 and the calmodulin binding domain (CaMBD) of nNOS. Using molecular docking and molecular dynamics simulation, we found that SUMO1 modification stabilizes the conformation of nNOS CaMBD, and helps maintain a conformation beneficial for nNOS catalysis. Analysis of the polar contacts and hydrogen bonds, and the root mean square derivation results showed that R726 and R727 of CaMBD formed polar contacts or high occupancy hydrogen bonds with SUMO1. Correlation factor analysis and free energy calculations showed that the W716, L734, F740, M745, and F781 residues were also involved in the SUMO1/CaMBD interaction in an orientation-dependent manner. The potential inhibitor binding pocket of SUMO1, aimed at disrupting SUMO1/CaMBD binding, was detected from the virtual screening results. Our *in silico* studies revealed that interfering with the non-bonded interactions of SUMO1/CaMBD would blocked nNOS SUMO-ylation and subsequent hyperactivation. This work provides novel structural insight into the functional regulation of nNOS by post-translational SUMO1 modification, and provides suggestions for the design of drugs targeting nNOS hyperactivation.

## Introduction

Neuronal nitric oxide synthase (nNOS), an enzyme constitutively expressed and of high levels in the mammalian brain and skeletal muscle, catalyzes the conversion of L-arginine to nitric oxide. Previous studies have suggested that nNOS is required for learning and long-term memory (Kelley et al., 2009, 2011; Pavesi et al., 2013; James et al., 2015), as well as skeletal muscle contraction (Ito et al., 2013; Baldelli et al., 2014). However, excessive activation of nNOS in the neurons results in oxidative and nitrosative stress, which is associated with neuronal loss in various neuropsychiatric disorders including hypoxic-ischemic encephalopathy (Yu et al., 2008; Yin et al., 2013; Favié et al., 2018), ischemic stroke (Zhou et al., 2010), and traumatic brain injury (Qu et al., 2020). Therefore, elucidating the structural basis for the regulation of nNOS catalytic activity is important for understanding its physiological and pathophysiological significance.

In terms of structure, functional nNOS occurs as a homodimer. Each monomer mainly consists of a PDZ domain, an oxygenase domain (heme domain), a cross-linked helix referred to as the calmodulin-binding domain (CaMBD) owing to Ca^2+^/calmodulin binding, and a reductase domain (Zhou and Zhu, 2009). The reductase domain contains two sub-domains: the flavodoxin-like domain (residues 755–935) and the flavin-adenine-dinucleotide (FAD)-binding domain (ferredoxin reductase type, residues 990–1237), which provide the binding site for flavin mononucleotide, FAD, and nicotinamide adenine dinucleotide phosphate (Roman and Masters, 2006). Although the complete structural model of nNOS is unknown, the crystal structure of each domain has been reported previously (Zhang et al., 2001; Garcin et al., 2004; Piazza et al., 2012; Li et al., 2014). Previous studies have built an nNOS dimer model encompassing the oxygenase domain, CaMBD (referred to as nNOS CaMBD in this article, if not specially mentioned), and part of the reductase domain (residues 299–951), based on the existing ethical data (Astashkin et al., 2010). We have referred to the partial reductase domain as the FAD-binding domain (residues 755–951), since it contains the critical residues for FAD binding. In this nNOS dimer model, the FAD-binding domain in one chain interacts with the heme domain in another chain, and together with the remaining part of the heme domain, it forms the FAD-binding site. Although this theoretical model partially clarifies certain molecular functions like the synthase activity of nNOS, it lacks structural insight on the effect of covalent modifications on nNOS catalysis.

Post-translational modifications including phosphorylation, acetylation, S-nitrosylation, and SUMO-ylation, differentially regulate nNOS activity (Komeima et al., 2000; Rameau et al., 2007; Bansal et al., 2008; Watanabe and Itoh, 2011; Du et al., 2020). The enzymatic activity of nNOS is upregulated by S1412 phosphorylation, and downregulated by S847 phosphorylation and S-nitrosylation (Komeima et al., 2000; Rameau et al., 2007). Previous studies have revealed that nNOS is covalently modified by a small ubiquitin-like modifier 1 (SUMO1) *in vitro*, and in the hippocampal and cortical neurons (Watanabe and Itoh, 2011; Du et al., 2020). We further found that long-term neuronal activity promotes SUMO1 conjugation to K725 and K739 on nNOS CaMBD, and thereby facilitates nitric oxide production, nNOS S1412 phosphorylation, and downstream signaling events (Du et al., 2020). SUMO-ylation and calmodulin-binding occur in the same domain (CaMBD); and calmodulin-binding upregulates catalytic activity of nNOS by triggering nNOS conformational change (Rameau et al., 2007; Salerno et al., 2013). Therefore, we assumed that SUMO1 binding affects nNOS conformation similar to the way calmodulin-binding affects nNOS. Further, we assumed that the binding of SUMO1 to nNOS CaMBD triggers a global conformational change in nNOS, and stabilizes and upregulates the catalytic activity of nNOS.

In this study, to further elucidate the binding characteristics of SUMO1 and CaMBD, we built *in silico* models of SUMO1 docked with nNOS, and evaluated the interaction characteristics between SUMO1 and CaMBD through molecular dynamics simulations. Analysis of the molecular dynamics simulation results of the models suggest that the binding of SUMO1 to nNOS stabilized not only CaMBD but also the nNOS FAD-binding domain, which benefits catalysis. Based on these *in silico* models, we also carried out a virtual screening to look for several potential inhibitors of nNOS SUMO-ylation in a putative approach. These models provide novel structural insights into the functional regulation of nNOS by SUMO1 at the atomic level, and provide suggestions for the design of drugs targeting nNOS hyperactivation, which would be used for the treatment of neuropsychiatric disorders.

## Results

### The Surface of nNOS CaMBD Offers a Potential Docking Region for SUMO1 Interaction

**FIGURE 1:**
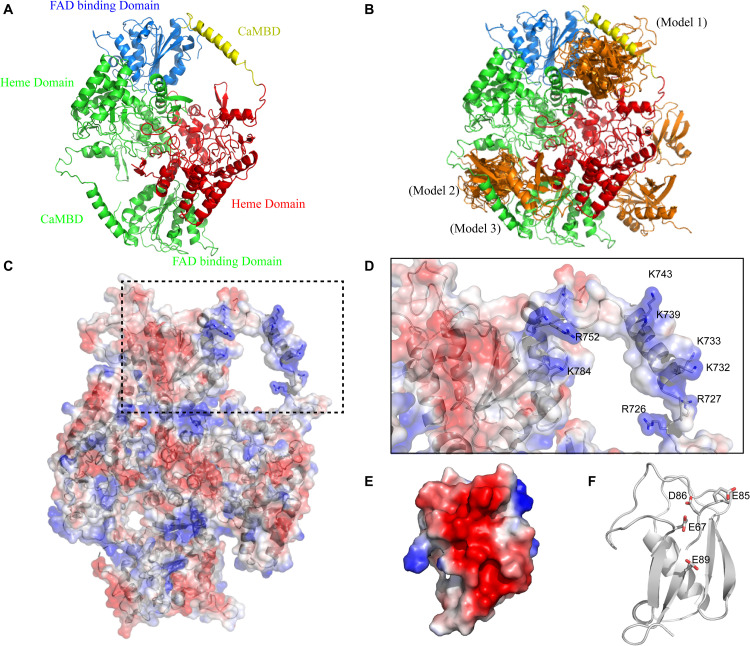
**(A)** Domain presentation of the nNOS homodimer (partial). The FAD-binding domain in one nNOS is shown in blue, CaMBD in yellow, and the remaining heme domain in red. The symmetrical parts are all shown in green. **(B)** Clustering results of SUMO1/nNOS docking. The top 15 results (with Z-DOCK scores ranged from 1046.102 to 1177.664) are shown in orange and the results extracted for Models 1, 2, and 3 are labeled. **(C)** Vacuum electrostatics calculations of nNOS (SUMO1-free) and the nNOS secondary structure are shown in the transparent vacuum electrostatics surface. Red/white/blue indicates a negatively/neutrally/positively charged surface, respectively. **(D)** Features of CaMBD are in the dash square and the important lysine/arginine residues in the formation of the positively charged surface are labeled. **(E)** Vacuum electrostatics calculation of SUMO1. **(F)** Important glutamate/aspartate residues in the formation of the negatively charged surface are indicated with sticks.

Firstly, we generated theoretical SUMO1/CaMBD models using Z-DOCK, based on the existing partial atomic structure of the nNOS homodimer, to show the docking of SUMO1 on nNOS surface (Figure 1A). Among the 100 Z-DOCK outputs, the top 15 conformations ranked based on their Z-DOCK scores showed that the CaMBD region of nNOS was favorable for SUMO1 docking (Figure 1B); SUMO1 was seen in close contact with CaMBD in 12 out of 15 conformations. To determine the reason behind the preference of SUMO1 for CaMBD, we performed vacuum electrostatic surface calculations representing the charge distribution on the surfaces of nNOS (Figures 1C,D) and SUMO1 (Figures 1E,F). As shown in Figures 1C,D, the surface surrounding CaMBD, including residues K784, R752, K743, K739, K733, K732, R727, and R726 exhibited great positive charges. Conversely, SUMO1 has a negatively charged surface produced by E89, E67, D86, and E85 residues (Figures 1E,F). Since the charges on these two surfaces are different, they attract each other through intermolecular electrostatic interactions, which form the basis for SUMO1/CaMBD non-bonded interactions. These docking results together with the vacuum electrostatic surface calculation results suggest that SUMO1 interacts with CaMBD via non-bonded interactions in addition to serving as a covalent modifier of the nNOS SUMO-ylation process.

**FIGURE 2:**
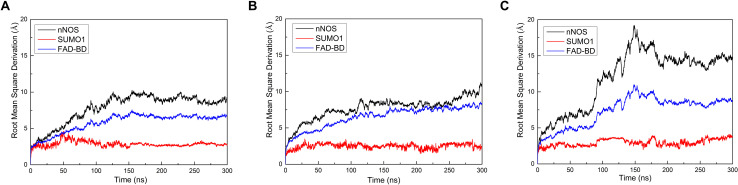
Root mean square derivation (RMSD) curves vs. simulation time for **(A)** Model 1, **(B)** Model 2, and **(C)** Model 3. The black/red/blue curves represent the RMSD curves of nNOS global structure/SUMO1/FAD-binding domain, respectively.

Based on the orientations of docked SUMO1, the conformational outputs of CaMBD/SUMO1 were clustered into three groups. One representative conformation was extracted from each group, and named Model 1, Model 2, and Model 3, respectively. Molecular dynamics simulations were performed to test the stability of these models. The root mean square derivation (RMSD) calculation (Figures 2A–C) showed that all three models reached equilibrium after approximately 300 ns. In each model, SUMO1 showed the least RMSD of approximately 2.5 Å, suggesting that SUMO1 maintains its original conformation throughout the simulation process. The FAD-binding domain, which is critical for nNOS catalysis, showed a terminal RMSD of 7–8 Å at 200–300 ns in all three models. This suggests that the FAD-binding domain undergoes a conformational change during the first 100 ns and reaches a slightly different conformation from the initial input. Although the RMSD results showed that nNOS reached equilibrium after 300 ns in all three models, the terminal RMSDs and RMSD curves were quite different from each other. In particular, the RMSD value of nNOS in Model 1 continued to rise between 0 and 150 ns and remained stable at a maximum value of 8–9 Å. In Model 2, the RMSD curve was similar to that of Model 1, except for a slight oscillation observed at the end of the simulation process. Model 3 showed an extremely large oscillation between 0 and 80 ns and reached equilibrium at 300 ns with a terminal RMSD of 15 Å, suggesting the occurrence of a large conformational change during the first 80 ns, resulting in a large difference between the terminal conformation and the initial conformation in Model 3. Since the RMSD values of all models showed only about 2–3 Å oscillations after 300 ns of simulation, we extracted the trajectory during the last 10 ns (290 to 300 ns) of all three simulation records and performed post-simulation analysis and calculated the essential parameter reflecting the characteristics of the CaMBD/SUMO1 interaction. The different terminal RMSD values, however, indicate that the allosteric regulation of nNOS, induced by docking different orientations of SUMO1, were totally different.

### SUMO1 Stably Binds to CaMBD Through Polar Contacts and Hydrogen Bonds

**FIGURE 3:**
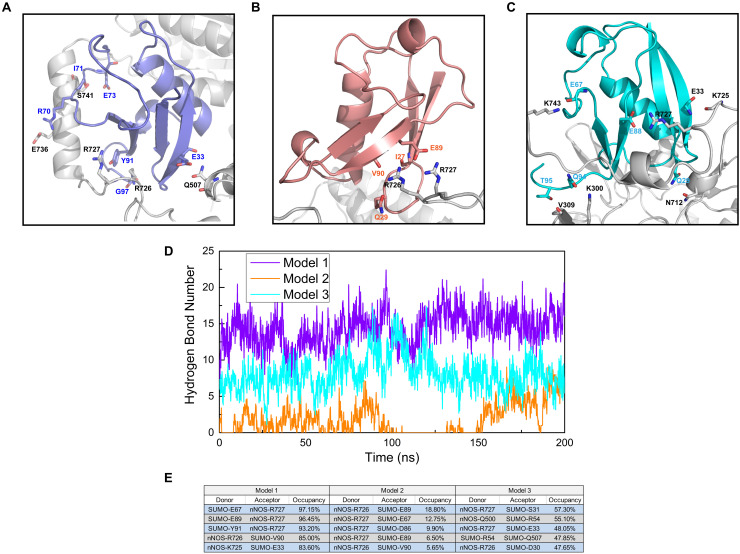
Residues involved in the intermolecular polar contacts (SUMO1/CaMBD) of **(A)** Model 1, **(B)** Model 2, and **(C)** Model 3. The nNOS residues are shown in black while the SUMO1 residues are shown in violet/orange/cyan, respectively. **(D)** Hydrogen bond number statistics of Models 1, 2, and 3 (vs. simulation time). **(E)** Top 5 hydrogen bond occupancy details of Models 1, 2, and 3. The donors and acceptors are listed.

We performed a polar contact study and analyzed the hydrogen bond statistics to identify the key interactions in SUMO1/CaMBD docking and determine the differences in the RMSD curves and values of the three models. From the analysis of polar contacts of terminal structures using molecular dynamics simulations, we found that the three models exhibited quite different intermolecular polar interactions (Figures 3A–C). In both Models 1 and 2, R726 and R727 were detected participating in the formation of intermolecular polar contacts. In Model 1, E736 of CaMBD also established a polar contact with R70 of SUMO1. Interestingly, the neutral residues of Model 1, including Q507 and S741, also established polar contacts with SUMO1; this was not seen in Model 2. In Model 2, except for R726 and R727 of CaMBD, no other residues showed polar contacts with SUMO1. The presence of fewer interaction sites in Model 2 results in terminal oscillations of the RMSD curve. In Model 3, the intermolecular polar contacts of R726 of CaMBD were not detectable, and only R727 and K743 were found to bind to charged residues. It is worth noting that R726 and R727 are located in the structurally unstable helix of CaMBD. This explains the instability of nNOS in Model 3 during the first 150 ns of simulation since there were no sufficient contacts between SUMO1 and CaMBD in Model 3 at that period.

In addition to polar contacts, hydrogen bonds also play critical roles in protein-protein interactions. To further investigate the intermolecular hydrogen bonds in SUMO1/CaMBD interactions, we performed hydrogen bond statistics with a cutoff distance of 3.5 Å and a cutoff angle of 35°. The total number of hydrogen bonds and the most important hydrogen bonds with the top five highest occupancies are listed in Figures 3D,E, respectively. Model 1 exhibited the highest number of hydrogen bonds among all three models. In contrast, the least number of intermolecular hydrogen bonds were detected in Model 2 (maximum occupancy was less than 30%). In Model 3, approximately 5–10 hydrogen bonds were detected during the entire simulation process, and the top 5 hydrogen bonds showed 47–58% occupancy. Therefore, in Model 3, the hydrogen bonds stabilize the SUMO1/CaMBD complex, thereby enabling the terminal RMSD values to attain equilibrium.

These data suggest that Model 1 has the most stable interacting conformation among all three models, owing to the maximum number of polar contacts and high-occupancy hydrogen bonds. Model 2 exhibits the least stability with poor polar contacts and weak hydrogen bonds. However, the polar contact and hydrogen bond statistics mainly focus on charged/polarized side chain residues or residues with hydrogen bond donor/acceptor, and more comprehensive data are required to find the fundamental reasons for the different binding orientations of SUMO1 in the three models, taking into account the contribution of the main chain and neutral residues.

### Structural Flexibility of CaMBD Decreases Upon Intermolecular Communication With SUMO1

**FIGURE 4:**
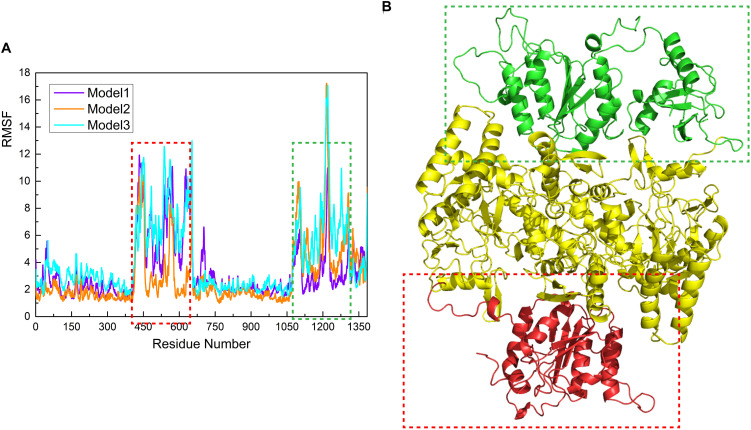
**(A)** Calculation of root mean square fluctuation (RMSF; Å) for the residues in the nNOS/SUMO1 interface in each model. The RMSF values (Å) of Models 1, 2, and 3 are shown as a violet/orange/cyan curve, respectively. **(B)** Structural presentation of green/red squared residues in nNOS. The green/red colored model represents green/red squared residues in **(A)**.

To investigate the global structural characteristics of the SUMO1/CaMBD interaction in all three models and calculate its flexibility in each model, root mean square fluctuation (RMSF) calculations were carried out and the RMSF values (by residues) of all three models were computed (Figure 4A). Since only one SUMO1 molecule was introduced to the nNOS homodimer, the SUMO1-free side of nNOS served as a perfect control to show the regulation of the CaMBD global structure by SUMO1. In the RMSF curves (Figure 4A), the residues with large RMSF values are marked with dashed squares (Figure 4B). The large RMSF values indicate that the most flexible regions of nNOS are CaMBD and the FAD-binding domain. The high flexibility of CaMBD can be attributed to the high random-coil percentage in its structure. Interestingly, in the SUMO1-bound side (green square in Figure 4B), we found that the average RMSF showed a slight decrease (about 1–2 Å) compared to the SUMO1-free side (red square in Figure 4B). This indicates that the binding of SUMO1 to CaMBD reduces its flexibility to some extent. The RMSF curves in the cyan/red squares were similar, but not 100% identical to each other, suggesting that SUMO1 does not behave the same in the simulation process of the three models. Essentially, the more polar contacts/hydrogen bonds in the model, the lower the RMSF value of the curve.

### Correlation Factor Analysis of the Global Residue Interactions in the SUMO1/CaMBD Complex

**FIGURE 5:**
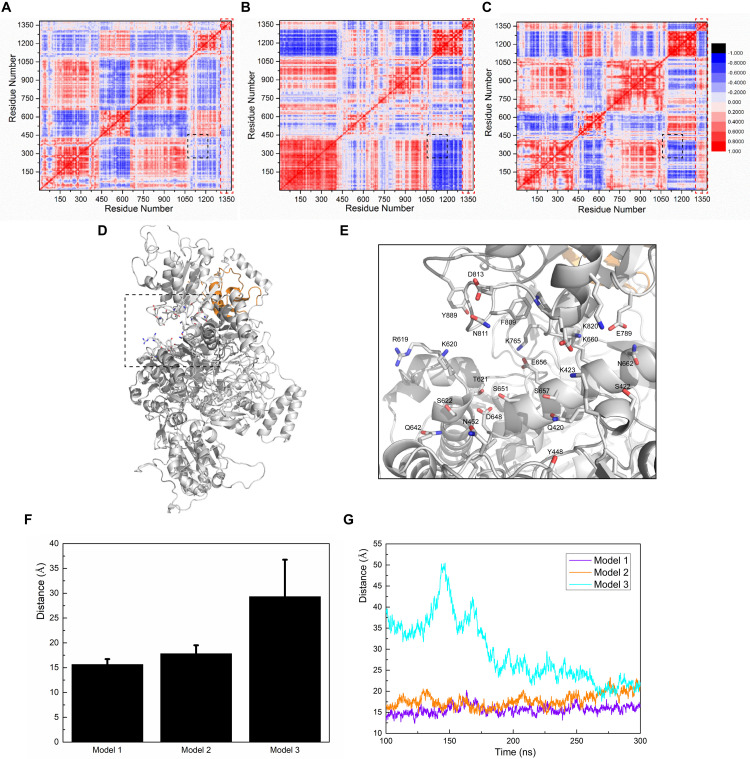
Calculations of correlation factors for **(A)** Model 1, **(B)** Model 2, and **(C)** Model 3. Highly correlated/anti-correlated residues are shown in red/blue, respectively. The legend is presented on the right side of **(C)**. The black square part and the red square part represent the CaMBD/SUMO1 related correlation factors discussed in the main text. **(D)** Presentation of the pocket constituents of Model 1, determined by the CASTp algorithm. **(E)** Featured conformation of the black square in **(D)**; residues marked by CASTp are drawn in stick style. **(F)** The average distance between the residue pair Q420-K820, and the standard derivation, calculated from the trajectories. **(G)** Depiction of the distances between the residue pair Q420-K820 vs. time, in the three models.

We calculated residue-to-residue correlation factors that reflect the vibration styles of each residue in both nNOS and SUMO1 (Figures 5A–C). The correlation factor figures of all three models showed a similar, but slightly different distribution of correlation factors. In particular, the black square part (numbers 1080–1210 to numbers 223–370, corresponding to residues 724–854 in nNOS and residues 521–671 in nNOS, respectively) of Model 1 was almost neutral, with little negative value, indicating that these two residue groups may not show any global correlation or anti-correlation changes. However, in Models 2 and 3, the black square part turned significantly negative and the two residue groups moved away from each other, suggesting the presence of some irresistible forces which triggered global conformational change. There is a high possibility that the SUMO1 orientations of Models 2 and 3 are less favorable compared to that of Model 1, thereby failing to render the intermolecular interaction network strong enough to hold the two residue groups together.

To further investigate this neutral-correlation region, we introduced the CASTp algorithm to test the stability of the pocket adjacent to the FAD-binding site of nNOS. Since the pocket formed by the two residue groups was adjacent to the FAD-binding site, their stability probably affected FAD binding. Additionally, they may also be involved in the regulation of nNOS catalytic activity. As expected, CASTp showed the pocket formed by the two groups of residues in Model 1 (Figures 5D,E), but failed to do so in Models 2 and 3. To obtain the exact data for comparison, the distance between Q420 (Cα atom) and K820 (Cα atom) was measured in all three models, as a reflection of the “mouth” size of the pocket (Figures 5F,G). As shown, the “mouth” size in Models 1 and 2 stayed at 15 Å and 20 Å, respectively, suggesting that the pocket was stable. In Model 3, the arginine binding pocket was not as close as that in Model 1 or 2 at 100–200 ns; however, the binding of SUMO1 to CaMBD stabilized the nNOS domains, prevented an increase in the RMSD of each domain, and held the FAD domain in its original position at the atomic level. At 300 ns, this binding reduced the distance between Q420 and K820 to approximately 20 Å. Distance measurement suggests that the FAD-binding domain did not move away during simulation, as such movement would have caused the protein to fall apart and destroyed any pocket in the protein. In contrast, Models 2 and 3 did not show any detectable pocket, indicating that the binding orientation of SUMO1 largely affects the conformation of the FAD-binding domain of nNOS. This suggests that, in Models 2 and 3, unlike in Model 1, the conformation of the FAD-binding domain and the nearby residues that interact with the FAD-binding domain in nNOS are stable. However, they are not found in their most suitable “pose” for FAD binding. Collectively, these results suggest that the SUMO1 binding orientation of Model 1 is theoretically better than that of Models 2 and 3. Model 1 better reflects the structural character of the SUMO1/CaMBD interaction during the SUMO-ylation of nNOS. Models 2 and 3 are also possible interaction models, yet not the best conformation reflecting the induced nNOS when SUMO1 binds to CaMBD.

### Free Energy Calculation Provides Residual Insight Into the SUMO1/CaMBD Interaction

**FIGURE 6:**
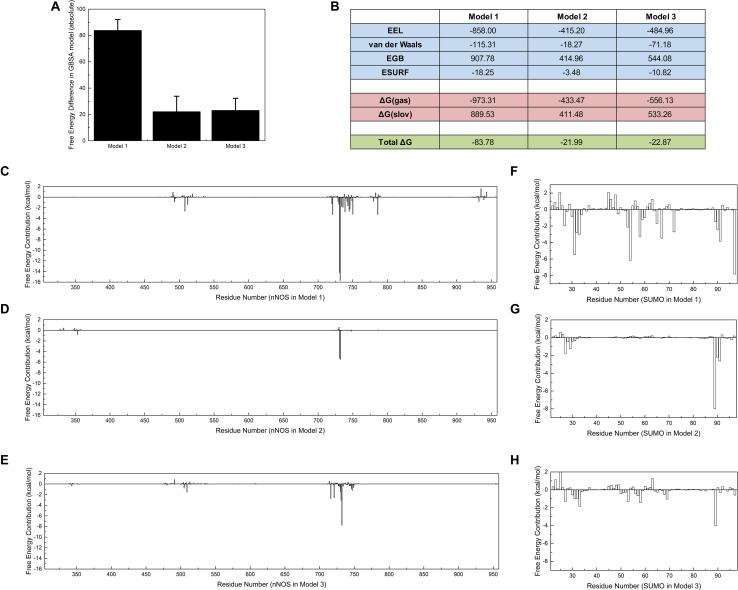
Free energy calculations and decompositions for each model. Absolute binding free energies of Models 1/2/3 are listed **(A)** with standard deviations, **(B)** with components of free energy in the calculation. **(C–E)** Energy decompositions of the nNOS part in Models 1, 2, and 3 are shown, respectively. **(F–H)** Energy decompositions which reflect the contribution of SUMO1 are shown. All units are in kcal/mol.

Polar contact analysis, hydrogen bond statistics, and correlation factor analysis provide general descriptions of the roles played by a single residue or a residue group in the SUMO1/CaMBD interaction. However, certain critical hidden residues in the SUMO1/CaMBD interaction remain elusive because only part of the residue characteristics were considered during these calculations. Some key contacts such as van der Waals interactions can be studied by binding free energy calculations. To achieve this, the binding free energy of the three models in an inclusive solution was calculated. In this calculation, SUMO1 was regarded as the ligand and CaMBD was regarded as the receptor. Model 1 showed the largest decrease in binding energy (Figure 6A), indicating that the orientation of SUMO1 in Model 1 was energetically more favorable compared to that in Models 2 and 3, whose decrease in the binding free energy was small. Electrostatics (EEL, related to polar contact) contributed to most of the binding energy in Model 1, almost twice as much compared to that in Models 2 and 3 (Figure 6B). This is consistent with the findings of the polar contact analysis. The contribution of van der Waals was more significant in Model 1 compared to Models 2 and 3, indicating that van der Waals interactions are critical in Model 1.

Further, to point out more key residues in the SUMO1/CaMBD interaction, the binding free energy results were decomposed into each residue (Figures 6C–H). As expected, the results showed that the most energetically critical residues in CaMBD were R726/R727. Model 1 showed several unnoticed critical residues: W716, L734, F740, M745, and F781, which contributed more than 2.5 kcal/mol of binding free energy. However, the contribution of these residues was not detectable in Models 2 or 3, suggesting that the orientation of SUMO1 plays an important role in the solid binding of CaMBD/SUMO1. The residues in SUMO1 contribute differently to each model. Interestingly, the residues contributing most to the binding free energy of Model 1 were not E89, E67, D86, or E85, but R55, S31, and Y92. However, in Models 2 and 3, the contributions of R55, S31, and Y92 were extremely low. Although S21 and Y92 did not possess any charged side chains, the van der Waals interactions of S31 and Y92 contributed most to the binding free energy of Model 1; this was not seen in Models 2 and 3. These free energy decomposition results reveal that the residues that formed polar contacts/hydrogen bonds, and those that contributed to van der Waals interactions were both critical in the SUMO1/CaMBD interaction.

### The Negatively Charged Surface of SUMO1 Is a Potential Target Site for Drug Development Disrupting SUMO1/CaMBD Interactions

**FIGURE 7:**
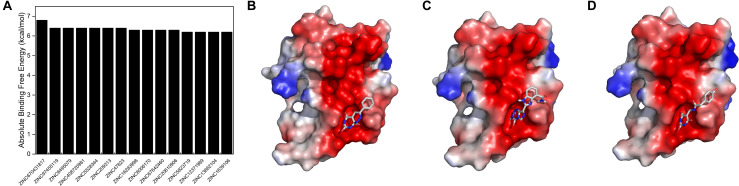
**(A)** Absolute binding free energies of the top 15 candidates from the virtual screening results. **(B–D)** The docking models of SUMO1 with ZINC670431817, ZINC97455119, and ZINC6495079, respectively.

To reveal the pharmaceutical significance of the SUMO1/CaMBD binding interface, we performed a virtual screening of 331,889 candidates using the ZINC15 database. The negatively charged rich surface of SUMO1 was set as the target binding site. The screening results were arranged based on the absolute binding free energy, calculated using Autodock Vina. We obtained the top 15 outputs and found that they all showed a decrease of >6.2 kcal/mol of absolute binding free energy (Figure 7A). Binding conformations of the top three candidates (Figures 7B–D) indicate the presence of a potential “pocket” next to E89 of SUMO1, since the partial ring sub-structure of all the three candidates show a good structural fit with the pocket formed by E89. Collectively, this SUMO1/CaMBD interface serves as a novel target site for the development of drugs inhibiting nNOS SUMO-ylation.

## Discussion

In this work, we generated three SUMO1/CaMBD binding models and performed polar contacts analysis, hydrogen bond statistics, RMSFs, correlation factor analysis, and free energy analysis on them. Together, they not only represent the most stable/detailed binding models for the structural interactions between SUMO1 and nNOS CaMBD, but they also point out the spatial characteristics of the nNOS SUMO-ylation site, which provide further insights into the study of protein SUMO-ylation. These results also provide hints on the design and improvement of specific nNOS inhibitors targeting nNOS SUMO-ylation, thereby contributing to the discovery of new treatments for neuropsychiatric disorders caused by nNOS hyperactivation.

Generally, SUMO-ylation is a post-translational modification that regulates protein activity, stability, cellular localization, and signaling pathways. The alterations in protein SUMO-ylation are observed in the regulations of neuronal and synaptic functions (Henley et al., 2018, 2020). However, several studies have suggested that SUMO proteins act as structural regulators via non-covalent interactions with certain proteins (Elrouby, 2014). This work showed that SUMO1 interacts with CaMBD through a non-bonded interaction, providing a novel way to analyze the binding between nNOS and SUMO1. The three models, especially Model 1, suggested the possibilities in the binding of SUMO1 to nNOS and pinpointed the critical residues in the nNOS/SUMO1 interaction. Although SUMO1 has been known to covalently bind to target proteins like nNOS, other non-covalent interactions also exist. These non-covalent interactions lead to global conformational changes and further regulate the catalytic activity of nNOS. It is worth noting that these non-bonded interactions (polar contact, hydrogen bonds, and other interactions contributing to the binding free energy) aid in the recruitment of relevant enzymes prior to nNOS SUMO-ylation because the non-bond interactions, especially electrostatic interactions, are usually long-range ones (Shashikala et al., 2019). They take effect prior/stronger to other structural contacts of protein binary interactions like SUMO1/CaMBD (Yang et al., 2019). This work also showed that SUMO1 binds to CaMBD with different stabilities, and in different orientations. The binding “pose” of SUMO1 in Model 1 showed the best polar contacts, hydrogen bond formation, and van der Waals interactions, and was the most favorable to depict the stable interaction between CaMBD and SUMO1. These non-bonded interactions stabilize FAD/NADPH binding, and thereby benefit nNOS catalysis.

Generally, the protein SUMO-ylation site is consistent with the residue sequence “Ψ-K-D/E-X” (Ψ is a hydrophobic residue and X is any residue) (Rodriguez et al., 2001). However, we found that several of the SUMO-ylation sites were not identical to the existing conserved sequence, suggesting that SUMO-ylation is regulated through certain unknown mechanisms and that the SUMO-ylated lysine residues have more identical characteristics compared to those in some similar “conserved sequences” (Zhao et al., 2014). In this case, K725 was SUMO-ylated, although it was not identical to any SUMO-ylation motifs identified in our previous work (Du et al., 2020). A possible explanation is that the spatial structure surrounding the SUMO-ylated lysine residues aid in the recognition of SUMO by the target protein and/or helps in the recognition/binding of SUMO-ylation-associated enzymes to the target proteins. Future studies underlying these mechanisms are warranted to obtain a clear understanding of protein SUMO-ylation.

This theoretical study of nNOS SUMO-ylation provides a new pathological outlook to nNOS inhibition in cognitive decline and myopathies (Kelley et al., 2009, 2011; Ito et al., 2013; Pavesi et al., 2013; Baldelli et al., 2014; James et al., 2015). Generally, the hyperactivity of nNOS produces excessive nitrogen oxide, which is toxic to neurons after acute brain injury (Yu et al., 2008; Zhou et al., 2010; Yin et al., 2013; Favié et al., 2018; Qu et al., 2020). Therefore, the downregulation of nNOS is important for neuroprotection. However, “direct” nNOS inhibitors targeting nNOS active sites may impose certain side effects since these inhibitors have poor nNOS specificity (Poulos and Li, 2017). Therefore, the side effects generally originate from the similarity between nNOS active sites and other L-arginine-related enzymes, which simultaneously bind to these nNOS inhibitors (Víteček et al., 2012). Thus, a more specific nNOS regulator that aims at other positions of nNOS is needed. Inhibition of nNOS SUMO-ylation is not as harsh as inhibiting nNOS itself. Therefore, this can be a possible treatment approach for diseases caused by nNOS hyperactivity. However, the challenge remains, because nNOS is not the only protein that has CaMBD, and SUMO1 has many other binding partners. Directly mimicking either CaMBD or SUMO1 can cause more specificity problems than expected.

This work provides a structurally novel design approach. The interface responsible for SUMO1/CaMBD binding is a potential target site for the design of nNOS SUMO-ylation inhibitors. Drug developers can test chemical compounds such as ZINC670431817, ZINC97455119, and ZINC6495079 *in vivo*, to determine their ability to inhibit nNOS SUMO-ylation and improve their affinity by altering their chemical structure. In addition, drug developers can also refer to the non-bonded Model 1 and design specific peptides by linking the residues involved in the SUMO1/CaMBD interaction, which are more specific, than just mimicking SUMO1 or CaMBD. This is a novel method for the development of drugs specifically inhibiting nNOS. It could give hints for the treatment of not only the neuronal diseases but also diseases of the muscle since the hypoactivation of nNOS splices could lead to disorders in skeletal muscle cells (Percival, 2011; Baldelli et al., 2014).

Collectively, our results not only show that SUMO1 docks to CaMBD and the nearby residues of nNOS, but also suggests that SUMO1 binding affects residues near the FAD-binding domain. The models in this study provide new structural insight into nNOS regulation and further benefit the design of drugs inhibiting nNOS SUMO-ylation.

## Materials and Methods

### Molecular Docking and Model Building

Neuronal nitric oxide synthase model building was accomplished by docking three existing NOS models (PDB: 1OM4, 1TLL, and 2LL6) with the nNOS sequence (*Rattus norvegicus*). Since no nNOS CaMBD structures were solved, homology modeling was used to build the final model. The final model was consistent with the previously reported nNOS model (Astashkin et al., 2010). All molecular docking experiments were performed using Z-DOCK (Pierce et al., 2014). Amino acids involved in the active site of nNOS-CaMBD were set as flexible residues. A genetic algorithm was used to minimize system energy and prepare docking conformations. A total of 100 possible substrate conformations were exported for cluster analysis. Conformations with maximum Z-DOCK scores were extracted and clustered. The representative conformations were used for subsequent molecular dynamics simulations.

### Molecular Dynamics Simulations

All molecular dynamics simulations (performed by the computers in Shanghai Tech University) were carried out using the AMBER 18 package (Case et al., 2018), utilizing the amber14sb all-atom force field parameters together with General Amber Force Field (GAFF) parameters (Wang et al., 2004; Maier et al., 2015). About 20 K^+^ and Cl^–^ ions were introduced to maintain an ionic strength of 100 mmol, and several K^+^ ions were introduced to neutralize the charge. Each system was explicitly solvated using the TIP3P water potential, inside a box of water molecules, with a minimum solute-wall distance of 10 Å. The protocol used for all molecular dynamics simulations is as follows: (1) the energy of the whole system was minimized to remove unfavorable contacts. Four rounds of 2500-steps of minimization were performed. In the first two rounds, the whole system was restrained except for water and ions; the minimization methods used in the first two rounds were steepest descent (SD) and conjugate gradient (CG), respectively. In the last two rounds, the whole system was unrestrained. The cutoff distance used for the non-bonded interactions was 6 Å. The SHAKE algorithm was used to restrain the bonds containing hydrogen atoms; (2) the energy-minimized structure was heated over 200 ps from 0 to 300 K (with a temperature coupling of 0.2 ps), while the atom positions of the protein were restrained with a small value of 10 kcal/(mol × Å^2^); (3) unrestrained equilibration of 200 ps was carried out for each system in the NPT ensemble (constant particle number, pressure, and temperature) with a temperature and pressure of 300 K and 1 bar, respectively, with a corresponding coupling of 0.2 ps. An integration step of 2 fs was used. (4) Finally, unrestrained molecular dynamics was carried out for 500 ns for the protein model. Other simulations followed the same protocol; (5) in the conformation output step, the average frame of the trajectory in the last 10 ns was exported for overall structural analysis.

### Fluctuations and Correlation Analyses

The root-mean-square fluctuation (RMSF) values of residues represents the measurement of the fluctuations and flexibilities of the Cα atoms of the protein backbone throughout the trajectory broken down by residues, in comparison to the average structures. The RMSF_*i*_ value of the Cα atom of each residue was calculated as follows:

$$R\,M\,S\,F_{i}=\sqrt{\frac{\sum_{t=1}^{T}{\left(r_{i}\,\left(t\right)-\left(r_{i}\right)\right)}^{2}}{T}}$$

Where T is the number of snapshots considered in the time trajectory, r_*i*_(t) is the position of the Cα atom of residue i at time t, and <r_*i*_> is the time-averaged position of the Cα atom of residue i.

The dynamic features of the protein and the extent of correlation of motions in different regions of the protein were assessed by calculating the cross-correlation coefficients or C(i,j), as follows:

$$C\,\left(i,j\right)=\frac{\left(\Delta \,r_{i}\times \Delta \,r_{j}\right)}{\sqrt{\left(\Delta \,r_{i}^{2}\,\Delta \,r_{j}^{2}\right)}}$$

Where Δr_*i*_ and Δr_*j*_ are the displacement vectors for Cα atoms of residues i and j, respectively, and the angle brackets denote the ensemble averages. In the present study, the correlation coefficients were averaged over the regions of the protein, and the resulting cross-correlation coefficients were presented in the form of a two-dimensional graph. In the present work, these structural analyses were performed using the CPPTRAJ module of the AmberTools package (Roe and Cheatham, 2013).

The binding pocket calculations in this work were completed on the CASTp website^1^ using computational geometry (Tian et al., 2018).

### Virtual Screening

All ligands were selected and extracted from the files in the ZINC15 library (Sterling and Irwin, 2015). The extracted ligands had molecular weights ranging from 200 to 300 Da and LogP ranging from −1 to 1 (neither too hydrophilic nor too hydrophobic). Only ligands marked as “agent” were selected in order to find the compounds available for further experiments. The Autodock Vina software package (version 1.1.2) (Trott and Olson, 2010) was used to perform the virtual screening after splitting the ligand data from ZINC15 into individual files (.pdbqt). By default, Gasteiger charges were applied to all residues of SUMO1, prior to screening.

## Data Availability Statement

Publicly available datasets were analyzed in this study. This data can be found in the RCSB Protein Data Bank (https://www.rcsb.org/) with the following accession numbers: 1OM4, 1TLL, 2LL6, and 3UIP.

## Author Contributions

Both authors listed have made a substantial, direct and intellectual contribution to the work, and approved it for publication.

## Conflict of Interest

The authors declare that the research was conducted in the absence of any commercial or financial relationships that could be construed as a potential conflict of interest.

## Footnotes

1 http://sts.bioe.uic.edu/castp/background.html
